# Supplementary material for: Genome-Wide Identification and Characterization of Bovine Fibroblast Growth Factor (FGF) Gene and Its Expression during Adipocyte Differentiation
Source: Int J Mol Sci. 2023 Mar 16;24(6):5663. doi: 10.3390/ijms24065663 (PMC10054561; doi:10.3390/ijms24065663)
Supplement: Supplementary file 1 [file ijms-24-05663-s001.zip › ijms-2253523-supplementary/Supplementary File/Additional file S5.pdf]

Motif 1

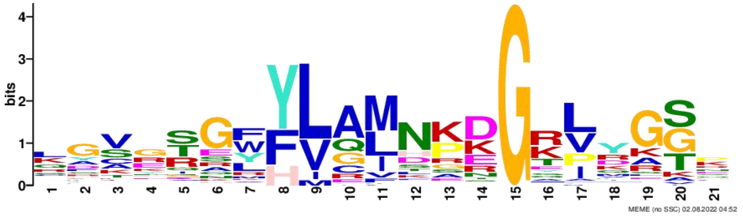

Motif 2

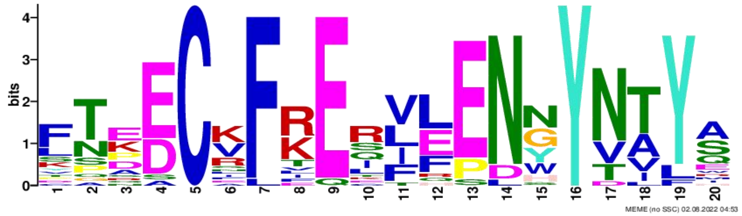

Motif 3

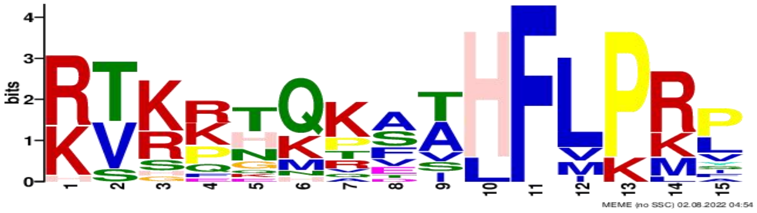

Motif 4

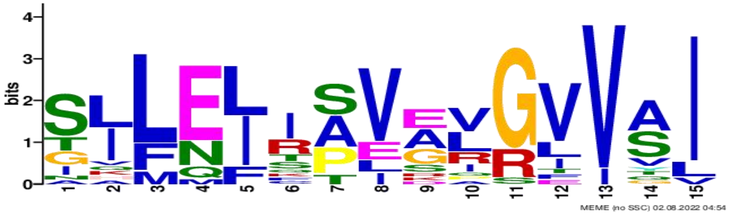

Motif 5

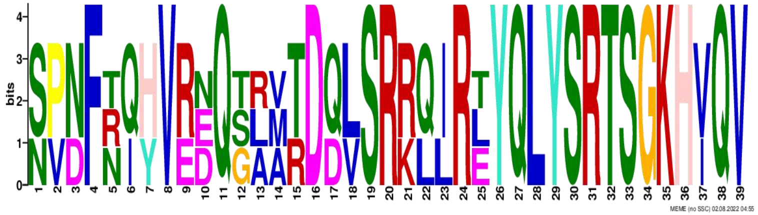

Motif 6

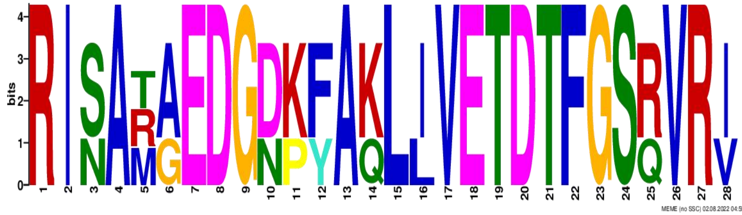

Motif 7

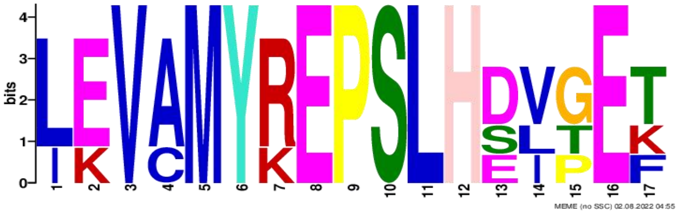

Motif 8

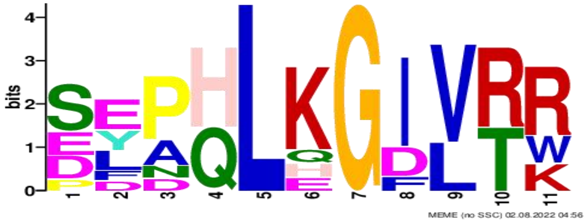

Motif 9

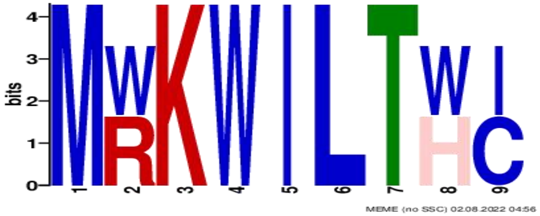

Motif 10

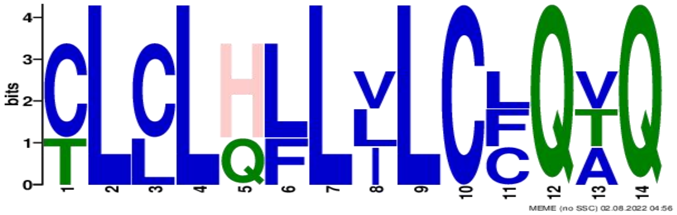

Amino acid sequence

Amino acid sequence
